# Multiple Sclerosis Patients and Disease Modifying Therapies: Impact on Immune Responses against COVID-19 and SARS-CoV-2 Vaccination

**DOI:** 10.3390/vaccines10020279

**Published:** 2022-02-11

**Authors:** Maryam Golshani, Jiří Hrdý

**Affiliations:** Institute of Immunology and Microbiology, Faculty of Medicine, Charles University, 128 00 Prague, Czech Republic; maryam.golshani@lf1.cuni.cz

**Keywords:** SARS-CoV-2, COVID-19, multiple sclerosis, DMTs, immunosuppression, vaccine

## Abstract

This article reviews the literature on SARS-CoV-2 pandemic and multiple sclerosis (MS). The first part of the paper focuses on the current data on immunopathology of SARS-CoV-2 and leading vaccines produced against COVID-19 infection. In the second part of the article, we discuss the effect of Disease Modifying Therapies (DMTs) on COVID-19 infection severity or SARS-CoV-2 vaccination in MS patients plus safety profile of different vaccine platforms in MS patients.

## 1. Severe Acute Respiratory Syndrome Coronavirus 2 (SARS-CoV-2)

The severe acute respiratory syndrome coronavirus 2 (SARS-CoV-2) causing coronavirus disease 2019 (COVID-19) has killed millions of people in a worldwide pandemic and has become a global public health emergency [1,2,3,4]. The virus initially originated from Wuhan, China on December 2019 and up to the 1 February 2022, about 379,298,536 confirmed cases with 5,693,522 deaths are officially reported by the governments of 225 countries to the coronavirus worldometers [5]. On the 1 February 2022, the World Health Organization (WHO) and Centers for Disease Control and prevention (CDC) estimated the fatality rate of COVID-19 disease to be between 0.00–1.63% which is higher for those over 50 years of age [6,7,8].

### 1.1. Ethiology of COVID-19 Disease

SARS-CoV-2 is an enveloped single-stranded positive-sense RNA virus which belongs to the β Coronavirus family. The 29.9 kb genome of the virus includes 13–15 open reading frames (ORFs), among them the amino acid sequences of seven conserved domains in the genomic ORF1ab, which are 94.6% identical to those from original SARS-CoV [3,4,9,10,11]. Bats of certain species are recognized as the natural host for a broad spectrum of CoVs. Having 90.4–100% amino acid identity with a coronavirus strain isolated from the Malayan pangolin, SARS-CoV-2 could be emerged from a possible recombination between viruses of bat and pangolin [12,13]. Spike protein (S), envelope protein (E), membrane protein (M) and nucleocapsid protein (N) are four major structural proteins of SARS-CoV-2. Moreover, the virus contains non-structural proteins which are not only essential in virus replication and assembly, but are also in viral pathogenesis process including modulation of early transcription regulation, gene transactivation, evasion of antiviral response, and immunomodulation [3,4,9,10,11,14].

### 1.2. Pathogenesis of SARS-CoV-2

Coronaviruses are usually the cause of respiratory infections ranging from the common cold to more severe diseases such as Middle East Respiratory Syndrome (MERS), SARS and recently emerged SARS-CoV-2 [1,9,14,15,16,17]. The viral RNA of SARS-CoV-2 is enveloped in a bilayer lipid crowned with S-proteins. 1273-amino acid S protein plays a key role in initiation, transmission and ongoing of SARS-CoV-2 infection. The S protein structure consists of two main domains: the N-terminal S1 domain (NTD) mediating adhesion of the virus to angiotensin-converting enzyme-related carboxypeptidase 2 (ACE2) expressed by the type II pneumocytes (vascular endothelial and alveolar macrophages and epithelial cells) in the human lung and the C-terminal S2 domain promoting the viral membrane fusion with the host cell membrane. The 424–494 amino acid subdomain of S1, receptor-binding domain (RBD), interacting with the peptidase domain (PD) binding site on ACE2 is the main target for neutralizing antibodies (Abs) to block SARS-CoV-2 entrance to host cells [7,8,9,11,12,13,14,15,16,17,18,19]. Upon binding to the receptor, the virus inters the host cell via receptor-mediated endocytosis where in the acidic environment, proteolytic cleavage of S protein into S1 and S2 subunits happens by a furin, cathepsin, TMPRSS2, or another protease. Upon S2-assisted fusion of the viral and cellular membranes, the viral RNA genome is released in the cytosol, where genomic replication and translation of the viral proteins take place. Eventually, interaction of structural proteins (S, E, and M) with the RNA genome packed in N proteins happens in endoplasmic reticulum-Golgi intermediate compartment (ERGIC) and virions are transported out of the host cell via an exocytic pathway [4,14].

Infection of pneumocytes by COVID-19 stimulates local inflammatory responses and induces the release of cytokines like tumor necrosis factor-α (TNF-α), transforming growth factor-β1 (TGF-β1), interleukin-1β (IL-1β), IL-6, and also numerous chemokines that act in recruitment of circulating leukocytes [3,4,14,17,18,19,20]. SARS-CoV-2 infection activates innate immune responses, plus antigen-specific cytotoxic T-cells directed against the virus and also B-cell responses with the ultimate production of neutralizing antibodies. In severe forms of COVID-19 infection, the ensuing inflammatory cascade may lead to the elevation of serum cytokines IL-2, IL-7, IL-10, granulocyte colony-stimulating factor (G-CSF), monocyte chemotactic protein (MCP), and TNF-α levels, which are called a cytokine storm. Although the immune system eliminates SARS-CoV-2 in most cases, vascular damage, hypercoagulation, hyperactivated inflammatory innate responses, and peripheral lymphopenia can result in multi-organ failure, acute respiratory distress syndrome (ARDS), and in some cases, death [1,3,4,11,14,17,18,20,21,22].

Neutralizing Abs, particularly anti-RBD antibodies, can play a role in the clearance of SARS-CoV-2 primary infection [9,20,21,23]. The higher ratios of anti-S1 or anti-RBD domains IgG antibodies compared to the nucleocapsid protein contribute to the milder COVID-19 disease showing the importance of these antibodies. On the other hand, studies show that regardless of immunosuppression, patients suffer from X-linked hypogammaglobulinemia and most patients with iatrogenic B-cell depletion can recover from COVID-19 infection. These findings indicate that although they have a significant role in response to primary infection, neutralizing antibodies are not strictly essential for recovery [14,20,21,22,24]. It is believed that T-cells play a critical role in the control and resolution of active SARS-CoV-2 infection, as there is an important association between SARS-CoV-2-specific CD4+ and CD8+ T-cells and disease severity. Patients with mild to moderate forms of COVID-19 infection have activated CD4+ T-cells, cytotoxic T-cells and follicular helper T (Tfh) cells, plus elevation in antibody-secreting plasmablasts, and the levels of IgM and IgG antibodies in blood. In contrast, in patients with severe disease, proportion of CD8+ cytotoxic T-cells expressing CD38 or double positive CD38 + PD-1+ is higher than in patients with the moderate course of disease and healthy controls indicating the hyper-activation of peripheral T-cells. Some features of the immune response, such as reduced expression of CD16 on neutrophils, monocytes and immature granulocytes, observed in the severe forms of COVID-19 infection, are similar to immune dysregulation found in sepsis. Moreover, some typical characteristics of an acute viral infection like activated T-cells and expansion of antibody-secreting plasmablasts have been indicated. Finally, reports show that there is a link between the levels of some chemokines and cytokines CXCL10, IL-6 and IL-10 in the blood and the severity of the disease [9,14,18,20,21,22,24].

### 1.3. Vaccine Platforms against COVID-19

Today, vaccination seems to be the most effective way to prevent COVID-19 infection, disease, or transmission [9,25]. By the end of February 2021, more than 40 countries and regions have been working on COVID-19 vaccine development and in total, 256 COVID-19 vaccine candidates have been developed based on different approaches, including live attenuated or inactivated vaccines (8.2%), non-replicating viral vector vaccines (13.3%), replicating viral vector vaccines (9.8%), recombinant protein-based vaccines (protein subunit vaccines (35.9%), virus-like particles (VLP)), and nucleic acid vaccines (DNA- (10.2%) and mRNA-based (12.1%) vaccines) (Table 1) [1,3,9,23,25,26]. As S protein is critical for the entrance of virus to the host cell, many COVID-19 candidate vaccines were designed based on the whole or a fragment of SARS-CoV-2 spike protein [1,3,11,23,27,28].

## 2. Multiple Sclerosis

### 2.1. Cause and Risk Factors

Multiple sclerosis (MS) is an adaptive and innate immune-mediated disorder defined as a chronic inflammatory autoimmune disease of the central nervous system (CNS). Pathology of MS is characterized by inflammation, demyelination, activation of microglia, proliferation of astrocytes and gliosis and different grades of axonal degeneration linked to oxidative stress and mitochondrial injury. Although the exact etiology of MS is still unknown, it is believed that some environmental, genetical, and epigenetic factors are playing a central role in induction and progression of the disease [29,30,31]. Globally, there are estimated number of 2.8 million people with MS and the prevalence of the disease was 35.9 per 100,000 population in 2020 which is expected to increase in the future [32,33].

### 2.2. Multiple Sclerosis and Infections

Patients with MS, especially those with the severe forms of the disease, have a higher risk for acquiring certain types of viral and bacterial infections. It has been shown that there is a link between bacterial and viral infections and greater chance of occurrence of relapses or pseudo-relapses in MS patients [34,35,36,37]. Additionally, almost all MS patients are under treatment with immunomodulatory or immuno-suppressive disease-modifying therapies (DMTs) to lessen disease activity, severity and to prevent or slow disease progression. These DMTs are classified as injectables agents (interferon-beta (IFN-β) and glatiramer acetate), monoclonal antibodies (natalizumab, alemtuzumab, ocrelizumab, rituximab and ofatumumab), and oral drugs (fingolimod, dimethyl fumarate, cladribine, teriflunomide, and ozanimod) with mechanisms of action including: lymphocyte depletion, disruption of lymphocyte replication, or alteration of lymphocyte trafficking [35,38,39,40]. DMTs, particularly B-cell depleting drugs (anti-CD20), also increase the risk of upper respiratory tract infections, urinary tract infections, opportunistic infections, infection-related hospitalization, and infection-related mortality rates in these patients [36,37].

### 2.3. COVID-19 Disease in MS Patients

The incidence of COVID-19 infection in patients with MS including suspected cases is reported between 1 to 11% with the mortality ratio of 1–4% (the general mortality ratio in 20 countries mostly affected by COVID-19 is 0.0–9.2%) [28]. According to the comprehensive cohort study performed by Coyle et al., history of treatment with anti-CD20 DMT therapies (such as rituximab and ocrelizumab), presence of comorbidities (congestive heart disease, diabetes mellitus, hypertension, chronic obstructive pulmonary disease, cardiomegaly, and obesity), older age, a longer disease course, higher disability, and progressive disease are risk factors associated with severe COVID-19 in MS patients [34,41,42,43]. The results of a similar study done by Sormani et al. show that the risk of hospitalization, intensive care unit (ICU) admission, and death after COVID-19 diagnosis of patients with MS were increased in cases with expanded disabilities and comorbidities. Moreover, the risk of hospitalization was higher in MS patients on anti-CD20 therapies than the patients on IFN or the healthy control group [44].

### 2.4. DMTs and COVID-19 Disease: Benefits-to-Risk Ratio

It should be considered that most of the MS patients on anti-CD20 therapy have the moderate to low mortality/morbidity risk to COVID-19 and they make an unremarkable recovery from the disease. This can be explained by the main role of innate immunity and T-cell responses in the clearance of SARS-CoV-2 (Table 2). On the other hand, B-cell and antibody responses do not seem to be essential for eliminating the primary infection, but are likely significant in elevation of secondary immune responses to prevent from reinfection in people infected in the past or infection in vaccinated ones [10,17,38,40,45,46,47,48,49]. Since ARDS or multi-organ failure is more likely to be caused by cytokine storm and overactivation of inflammatory responses to the virus than the virus itself, the moderate immunosuppression made by some DMTs having antiviral activities (like IFN-β and teriflunomide) or blocking excessive host immune responses may prevent severe COVID-19 infection complications. Some studies reported cases of MS patients on fingolimod (a sphingosine analogue) and tested positive for COVID-19 infection, but did not develop any symptoms or showed moderate to severe complications without any fatal case. At present, fingolimod is under investigation as a possible treatment for COVID-19-associated ARDS. Because of its antiviral activity, IFN-β is also under trial and it seems that it is even able to reduce the risk of COVID-19 infection in Italian MS patients [19,28,40,43,46,50,51,52]. Glatiramer acetate can shift a pro-inflammatory response to an anti-inflammatory response, which could be potentially advantageous in cases of COVID-19 infection. Moreover, glatiramer acetate blocks IFN-γ mediated activation of macrophages, which is thought to be significantly associated with ARDS. Teriflunomide decreases activation of immune responses without significant immunosuppression, which can prevent excessive host responses in cases of SARS-CoV-2 infection. It may also affect the replication of SARS-CoV-2 inside the infected cell. Therefore, presently, its antiviral activity and the mitochondrial enzyme dihydroorotate dehydrogenase (DHODH) inhibiting mechanisms are under consideration to prevent COVID-19 morbidity and mortality [34,40,43,49]. Because of its anti-oxidative, anti-inflammatory and cytoprotective effects, dimethyl fumarate can play a role in the control of COVID-19 infection via blocking SARS-CoV-2 replication and also expression of related inflammatory genes [49].

According to MS Global Data Sharing Initiative, patients on anti-CD20 antibodies are at risk of 1.5× more hospitalizations, 2.5× more intensive care unit (ICU) admissions, and 3× more use of mechanical ventilation in comparison to ones receiving other DMTs [1,35]. However, discontinuation of immunomodulatory therapy is not generally recommended by others, although caution should be considered with some treatments [28,41,50,53,54]. MS patients under treatment with IFN-β or glatiramer acetate (no risk of systemic infections) or the ones under treatment with DMTs like dimethyl fumarate, fingolimod, siponimod, teriflunomide, natalizumab, ocrelizumab, and rituximab (low risk), might continue their treatment without specific concerns, unless they develop significant lymphopenia (<500 lymphocytes/μL for dimethyl fumarate and <200 lymphocytes/μL with fingolimod) [28,49,50,54,55]. Although S1P modulators (fingolimod, siponimod, ozanimod, ponesimod) causes lymphopaenia by reducing the migration of lymphocytes from secondary lymphoid organs into the circulation, they do not increase the risk for COVID-19 infection [50,54,55].

According to a systematic review done by Barzegar et al., the highest rate of hospitalization was reported in patients with no DMT (42.9%), and patients on anti-CD20 therapies (29.2%), teriflunomide (20.6%) or fingolimod (14.7%) [25]. Both anti-CD20 agents and fingolimod act by disrupting the germinal center (GC) function in lymphoid tissue. GC is a location where: gene rearrangement and Ab class switching, affinity maturation and selection of high-affinity B-cell receptors or membrane-bound Abs take place with the help of Tfh cells. Eventually, memory B-cells and plasmablast clones leave the GCs for the peripheral blood where they produce high-affinity soluble IgG antibodies. Patients on no DMTs also had higher mortality and hospitalization rates because older patients or those in advanced terminal stages of MS are usually not treated with DMTs as the risk outweighs the benefit in these patients [56].

Although DMTs such as ocrelizumab and rituximab effectively reduce MS relapses by targeting B-cells and also reduce proinflammatory B-cell cytokines, prolonged use of these therapies (median duration 2.8 years) is rarely associated with severe infection [43,48,49]. In addition to the disruptive effect on GC formation in secondary lymphoid tissues, rituximab has a depletive effect on naive B-cells in the blood, lymphoid tissue and, to some extent, in the bone marrow, the risk of prolonged clinical courses and also hospitalization is higher in SARS-CoV-2 infected patients on rituximab [39,41,48]. Ocrelizumab has a minor impact on T-cell counts and is associated with mild to moderate viral infections (herpes viruses and involved the respiratory tract) and severe bacterial infections (pneumonia, urinary tract infections and cellulitis). Therefore, in patients who are under treatment with ocrelizumab, it is recommended to postpone the further infusions in case of active COVID-19 infection. However, it is shown that most MS patients treated with ocrelizumab have mild to moderate SARS-CoV-2 infections without the need for hospitalization [49,50,57].

Cladribine and alemtuzumab as B-cell depletion agents may enhance the risk of susceptibility to COVID-19 infection; thus their regular application or initiation of treatment should be considered carefully [49,53,54,55,58]. Alemtuzumab causes severe lymphopenia via decreasing the count of T- and B-cells for several months, thus increasing the incidence of infection following administration. Alemtuzumab is related to the occurrence of mostly mild to moderate infections, such as herpes zoster, oral herpes, upper respiratory-tract infection, tuberculosis, influenza, listeriosis, urinary-tract infection, and localized superficial fungal infections. Moreover, there are many cases of opportunistic infections including nocardiosis, cytomegalovirus, pyogenic granuloma, esophageal candidiasis, and spirochetal gingivitis are reported in patients treated with alemtuzumab. However, occurrence of the mild COVID-19 infection of a few MS patients treated with alemtuzumab demonstrates the relevant beneficial anti-inflammatory effect of this medication [43,49,50,54]. Cladribine mainly has a depleting effect on B-cells and T-cells (average of 50%) as CD4+ cells are reported to be more sensitive than CD8+ T-cells. Therefore, transient mild to moderate lymphopenia is a common adverse event. The effect of cladribine on innate immune cells such as erythrocytes, monocytes, NK cells, neutrophils, and platelets is minor. It is mandatory to perform clinical follow-up, standard laboratory tests, and screening for hepatitis B/C, HIV, and active tuberculosis before initiating cladribine therapy. When compared with the control group, the evidence rate of herpes zoster infections are also reported to be higher in MS patients treated with cladribine. Therefore, because of the higher risk of viral infections in 3–6 months after treatment with alemtuzumab and cladribine, it has been recommended to delay therapy with these drugs during the peak of the coronavirus pandemic [43,49,50,54,55].

Therefore, in the case of alemtuzumab, cladribine, rituximab, and ocrelizumab, due to the long immune reconstitution period (several months to years), MS patients who obtained their last dose within 6–12 months may still be immune-compromised. Accordingly, patients who continue treatment with these drugs may be at higher risk of COVID-19 infection. Young MS patients with no other comorbidities and lymphocyte count of >800/mm^3^ (WHO grade II) and are able to overcome viral infections. Patients with a lymphocyte count < 800/mm^3^ are at increased risk of COVID-19 infection and infection-related mortality (more than 50% risk) [40,50,54,55].

There are some reports showing that most of the MS patients on teriflunomide developed mild forms of SARS-CoV-2 infection including fever, gastrointestinal disturbances, fatigue and cough. Five patients out of 15 patients were hospitalized and two of them required oxygen therapy. Therefore, the authors suggest that teriflunomide therapy could be continued in patients with MS who develop an active COVID-19 infection [38,49,52,59,60]

Administration of natalizumab (a second line therapy) has been recommended by Brownlee as the safest substitute due to its low risk of systemic immunosuppression. Additionally, the North American Registry of MS patients reported a decreased risk of intensive care unit (ICU) admission and ventilation in patients treated with fumarate and natalizumab in comparison to untreated MS patients [40,41,49,59]. However, there is the theoretical concern that natalizumab can reduce lymphocyte trafficking in the lung and mucosa and so slightly enhance the risk of upper respiratory tract infections. As a licensed medication for Crohn’s disease, natalizumab decreases trafficking of lymphocytes to the gut. Therefore, this could be a concern as SARS-CoV-2 infects the gastrointestinal tract as well and approximately 3–4% of COVID-19 infected people develop diarrhea and shed the virus in the stool. Since natalizumab blocks immune surveillance of the CNS, patients treated with natalizumab can be in danger of major complications if they develop COVID-19 encephalitis [48,50,59].

Therapeutic protocols based on corticosteroid administration are not recommended because of their immune-suppression effects reducing the host ability to resist COVID-19 infection [50,54,55].

### 2.5. Effects of DNTs on Antibody Responses in COVID-19-Infected Patients

It is demonstrated that some DMTs can affect production of anti-SARS-CoV-2 antibodies upon infection in MS patients. According to some studies, MS patients who received anti-CD20 therapy (like ocrelizumab) showed reduction in the immunoglobulin production resulting in low or zero IgG antibody titers, but patients treated with teriflunomide, glatiramer acetate, dimethyl fumarate, and natalizumab showed IgG seroconversion [21,47,61]. Regarding ocrelizumab, there is a contradictory study indicating that patients suffering from COVID-19 and under ocrelizumab therapy had an IgG level within the normal range [57].

Gelibter and his colleagues reposted a case of COVID-19 infection in an MS patient treated with cladribine. The clinical symptoms including fever (<38 °C), cough, ageusia, anosmia, nasal congestion, and diarrhea were fully cleared after a few days without any need for hospitalization. However, the patient did not raise humoral responses and was negative for IgM and IgG anti-SARS-CoV-2 antibodies after infection [62]. A case of a patient with relapsing-remitting MS was also reported who developed COVID-19 pneumonia 2 weeks after treatment with cladribine. Despite of a severe lymphopenia, the patient had a moderate course of COVID-19 infection. In studies done by some authors, MS patients treated with cladribine experienced self-limiting COVID-19 disease with a favorable outcome, even in the presence of severe lymphopenia, no death or need for mechanical ventilation was reported. However, some patients did not develop any antibody response following infection [42,63,64,65,66]. Celius et al. also reported a patient who was under active treatment with cladribine for two years but experienced a mild COVID-19 infection and was able to raise an adequate antibody response against SARS-CoV-2 [66].

Flores-Gonzalez et al. reported a SARS-CoV-2-infected MS patient who was on treatment with ofatumumab for 42 months and was fully B-cell depleted. This patient was clinically asymptomatic and mounted adequate IgM and IgG antibody levels to the virus. The anti-SARS-CoV-2 IgG titers were detected three months after the initial positive serological testing [67].

Bollo and his colleagues reported two cases of MS patients infected with SARS-CoV-2; one patient treated with fingolimod who never developed respiratory difficulties during the hospitalization but showed limited humoral responses. Conversely, the teriflunomide-treated patient experienced a mild type I respiratory failure during the hospitalization but had an adequate SARS-CoV-2-specific antibody production [39]. In another case reported by Ciardi and colleagues, a teriflunomide-treated patient showed mild COVID-19 infection symptoms with lower percentages of fully differentiated CD4+ and CD8+ T-cells and a higher percentage of naive T-cells, suggesting that teriflunomide controls activation and the immunosenescence of T-cells [39].

An analysis of data from 28 countries on suspected or confirmed COVID-19 infection demonstrated that MS patients treated ocrelizumab or rituximab contributed to a notably higher risk of hospitalization and admission to the ICU and artificial ventilation than patients on other DMTs (including alemtuzumab, cladribine, dimethyl fumarate, fingolimod, glatiramer acetate, IFN-β, natalizumab, and teriflunomide) [34].

Differences or contradictory results reported in the above studies could be related to the sensitivity of the assays used to detect the specific anti-SARS-CoV-2 antibodies or distinct parts of SARS-CoV-2 spike protein detected by kits from different manufacturers (e.g., RBD, S1, S2, whole spike protein) [57].

### 2.6. Effect of DMTs on Immunity against SARS-CoV-2 Vaccination

As mentioned before, anti-RBD antibodies are more associated with the elimination of the primary SARS-CoV-2 infection than being required for recovery from COVID-19 infection; thus anti-CD20 therapies may decrease the efficacy of a vaccine against SARS-CoV-2 via depleting B-cells. However, T-cell responses induced by the vaccine are believed to play an essential role in protection against following SARS-CoV-2 infections. Accordingly, few studies reported the passive effects of some DMTs on cellular immune responses upon vaccinations (Table 3). The duration of treatment with certain DMTs may also be important [2,29,32]. There are some studies on the effect of anti-CD20 therapies on the COVID-19 vaccine efficacy among immunosuppressed patients. The results of these studies demonstrate that the level of anti-SARS-CoV-2 IgG, and also vaccine efficacy, were decreased in patients with malignancies, solid organ transplantation or inflammatory rheumatic diseases [68,69]. With regard to these facts, some authors suggest consideration of a 4–6 month time-window before and after vaccination due to the induction of rapid and prolonged (up to 24 weeks) B-cell depletion and attenuated humoral immune responsiveness by anti-CD20 antibodies and also differential kinetics of B-cells repopulation in immunocompromised patients. For instance, in the case of rituximab and ocrelizumab, repletion of immature/mature (naive) B-cell is completed within 12 and 18 months, respectively. Although CD19+ B-cell subsets, including memory (CD19+CD27+CD38low) B-cells are completely depleted during active treatment with ocrelizumab, amount of CD4+ and CD8+ T-cells is relatively stable. The repopulation time for cladribine and alemtuzumab is shorter, as the recovery of CD19 naive B-cells takes place within a median of 30 weeks and 6 months, respectively [25,27,34,35,70,71,72,73]. Accordingly, the National Multiple Sclerosis Society advises waiting at least 12 weeks after the last dose of B-cell-depleting therapies before vaccination. In these group of patients, measuring the CD19+ B-cells and CD19+ CD27+ memory B-cells count, at least every 3 months, is recommended before vaccination. The results of a study by Disanto and his colleagues show that there is a progressive increase in SARS-CoV-2 IgG levels with an increase in CD19+ B-cell count and time since last anti-CD20 antibody infusion. In treatment-naive patients or patients who were under treatment with first-line immunomodulators, vaccines should be given at least 2 weeks prior to administration of immunosuppressive drugs [25,34,35,70,71,73]. Currently, there are some studies demonstrating the efficacy of vaccine boosters on antibody responses in immuno-compromised patients on anti-CD20 therapies. Accordingly, administration of the third dose vaccination in MS patients, transplant recipients and patients with cancer increased the levels of humoral responses, even in patients who were sero-negative after the second dose due to anti-CD20 therapies. Therefore, these findings indicate the enhancer effect of additional COVID-19 vaccine dose on antibody levels in immunosuppressed group of patients [74,75,76,77,78,79,80,81].

Glatiramer acetate does not deplete lymphocytes; hence, it is unlikely to affect the protective immune responses to these vaccines in MS patients. Therefore, it seems that MS patients on glatiramer acetate will raise an appropriate amount of immune response to current COVID-19 vaccines [25,27,34,49,70,95].

As teriflunomide specially blocks the proliferation of auto-reactive lymphocytes and not other lymphocytes bearing TCR specific to foreign antigens (such as SARS-CoV-2 S protein), MS patients under therapy with teriflunomide will confer protective immune responses against the COVID-19 vaccines [49,95].

Since fumarates mainly act on downstream immune targets without depleting lymphocytes, in non-lymphopenic patients, they are unlikely to affect cellular immune responses elicited by COVID-19 vaccines. Therefore, the time-window for vaccination and checking the absolute lymphocyte counts before vaccination may be necessary to allow lymphocytic recovery [25,49,95].

Since S1P modulators (like fingolimod) trap T- and B-cells in the secondary lymphoid tissues resulting in reduced infiltration of these cells into CNS, MS patients on these therapeutical agents produce lower immune responses to SARS-CoV-2 vaccines [28,29,32]. Therefore, it will be beneficial to check the titers of anti-S protein-neutralizing antibodies after vaccination and decide whether it is necessary to administer a booster dose. In contrast to fumarates, interruption of S1P modulators treatment to maximize vaccine efficacy is not recommended due to the increased risk of severe MS rebound [95].

It is beneficial to administer COVID-19 vaccines at least 12 weeks post-ocrelizumab treatment and 4–6 weeks prior to the next dosing to increase vaccine efficacy [34,35,49,70,95].

Usually, rituximab therapy results in almost complete B-cell depletion, initiated 2 weeks after the infusion and lasting for 6–12 months. It is thus recommended to administer SARS-CoV-2 vaccines at least 3–6 months after rituximab last dosing to increase the vaccine efficacy. Post-vaccination checking of anti-S-neutralizing antibodies will be beneficial to evaluate the vaccine efficacy and possible need for a booster immunization [25,49,95,96].

The results of the study by McCarthy et al., evaluating the effect of alemtuzumab on vaccines, showed that vaccination within 6 months after therapy does not generate adequate immune responses. Therefore, the authors suggested waiting for vaccinations at least 6 months after alemtuzumab treatment [27,70]. MS patients treated with alemtuzumab may produce an attenuated immune response against SARS-CoV-2 vaccines if vaccination is performed at least 6 months after treatment. Moreover, it is advised to check the titers of neutralizing Abs after vaccination and to wait 4–6 weeks post vaccination before the next alemtuzumab administration to acquire adequate vaccine efficacy [49,95].

MS patients on ofatumumab should wait at least one month before and after the second vaccination to acquire the optimized level of protection against the vaccine. Since there is a 3 to 4 week interval between most SARS-CoV-2 vaccines doses, a couple of doses of atumumab must be skipped. In this regard, the single dose vaccine Ad26.COV2⋅S may be preferable for MS patients on ofatumumab, as it requires only skipping of one drug dose [95].

In order to improve the vaccine efficacy, the authors recommended a pause to siponimod therapy at least 7 days prior to administration of a vaccine and to resume siponimod (after up-titration) 2 or more weeks post-vaccination [70].

Regarding corticosteroids, it is generally advised to avoid administering live vaccines during treatment and until at least 4 weeks after discontinuing high-dose corticosteroids [70].

There is no study regarding the effect of ozanimod or oral cladribine on vaccine effectiveness generally [27,70]. MS patients treated with cladribine are recommended to postpone their next dose of cladribine until 4–6 weeks after vaccination. Patients who finished cladribine treatment and completed immune reconstitution (no lymphopenia) should produce full immune responses to SARS-CoV-2 vaccines [49,95].

### 2.7. SARS-CoV-2 Vaccines’ Safety in MS Patients

Eventually, COVID-19 will become endemic and thus serve as a seasonal risk, particularly for patients with immunosuppression. Although the rapid development of vaccines is promising, there is a concern about vaccine safety and efficacy in immunocompromised patients like MS patients on DMTs. Recently, the National MS Society published a guidance on COVID-19 mRNA vaccines regarding the vaccine safety in MS patients, advice for vaccination and guidelines for vaccination timing in relation to each DMTs to enhance the vaccine efficacy [2]. Furthermore, according to the current CDC recommendations, immunosuppressive patients can receive SARS-CoV-2 vaccines if they have no additional contraindications. However, these patients should be informed about the unknown vaccine safety profile and efficacy in immunosuppressed people. Finally, like other vaccines, COVID-19 vaccination during the very active phase of the disease or after administration of high doses of corticosteroids is not recommended [35].

Regarding SARS-CoV-2 mRNA vaccine safety, they do not contain the live virus and are not able to integrate with the human genome or cause COVID-19 infection. Both mRNA-1273 and BNT162b2 show similar effects, including injection-site pain and short-term fever symptoms, while severe adverse events are rare and comparable in vaccine and control groups. There is a concern about mRNA vaccines in MS patients, as these vaccines can induce type-I interferon responses, which are linked to inflammation and autoimmunity. However, injection with modified nucleotide mRNA COVID-19 vaccines may avoid this response [18,95,97,98]. The results of a study performed by Lotan et al. among BNT162b2 mRNA vaccinated MS patients on IFN-β, dimethyl fumarate, natalizumab, ocrelizumab, teriflunomide, fingolimod, cladribine, glatiramer acetate, or corticosteroids showed that 60% of cases who received just the first dose of the vaccine, 58.1% just after the second dose, and 9.7% after both doses reported new or worsening neurological symptoms [99].

Viral vector COVID-19 vaccines also do not replicate or integrate in the human genome and they do not cause COVID-19 nor Adenovirus infections. Adverse events in Ad26.COV2⋅S and ChAdOx1nCoV-19 are mainly reported as injection-site pain and short-term flu-like symptoms [18,95,100,101]. The use of ChAdOx1 nCoV-19 in MS patients should be concerned, as this vaccine has a potential to cause vaccine-related cases of transverse myelitis (TM) and other cases of immune-mediated and neurological events in recipients. However, no similar events are reported for Ad26.COV2⋅S, showing a slightly safer profile over ChAdOx1nCoV-19. Moreover, its single dose of injection makes it more compatible with DMT regimens. This frequency of immune-mediated adverse events caused by ChAdOx1nCoV-19 could be explained by the use of a Simian Adenovirus vector in this vaccine. Although not particularly related to MS patients or DMTs, there is general concern around the potential rare risk of venous thrombosis with thrombocytopenia in both the ChAdOx1nCoV-19 and the Ad26.COV2⋅S-receiving population. The number of cases of ChAdOx1nCoV-19 recipients (young ages) in several European countries have experienced disseminated intravascular coagulation and cerebral venous sinus thrombosis with thrombocytopenia. Generally, the stronger immune response in younger people is likely responsible for their susceptibility to vaccine reactions and increased risk of immune-mediated adverse events. Therefore, the concern raises towards the younger age group of MS patients, who will be at risk of post-vaccination immune-mediated reactions [95,101,102,103].

The DNA vaccines do not contain the live virus and do not interfere with or alter the host cell DNA. Similar to the previous results of trials investigating DNA vaccines against MERS coronavirus, the results of the Phase I clinical study on the COVID-19 INO-4800 DNA vaccine showed mild local and systemic events, with no report of any immunological or neurological adverse events [95,104].

Inactivated virus vaccines also have showed a safe profile in MS patients on different DMTs, as they do not replicate or cause COVID-19 infections in the host. The results of Phase-I/II trials for BBIBP-CorV indicated that all adverse reactions were mild to moderate, with no report of immune-mediated or neurological adverse events [95,105].

According to the American Academy of Neurology’s (AAN) 2019 guidelines, given the possible risk of infection associated with immunosuppression, live-attenuated vaccines are not generally recommended in MS patients who are under treatment with DMTs and those who recently took DMTs. At present, no safety data are available for COVID-19 live-attenuated vaccine MV-014-210 (Meissa) [49,95].

## 3. Conclusions

COVID-19 disease will eventually become endemic and thus a potential seasonal risk for immunosuppressed patients like MS patients. With regard to this fact, all MS patients with no additional contraindications are recommended to receive approved SARS-CoV-2 vaccines. However, SARS-CoV-2 live-attenuated vaccines are not recommended in these patients due to the risk of infection. COVID-19-infected MS patients under treatment with SP1 or anti-CD20 therapies may show a lower immune responses against the virus particles. Regarding the effect of DMTs on SARS-CoV-2 vaccination, MS patients under treatment with IFN-β and glatiramer acetate showed protective immune responses against SARS-CoV-2 vaccines. However, patients treated with cladribine, fingolimod, ocrelizumab or rituximab generated lower anti-Spike/anti-RBD IgG responses, but protective levels of CD4+ and CD8+ T-cell responses following vaccination with SARS-CoV-2 vaccines. Therefore, a time window for vaccination due to B-cell depletion and attenuated humoral responses by anti-CD20 antibodies is suggested.

## Figures and Tables

**Table 1:** Overview of the major SARS-CoV-2 vaccine strategies.

Vaccine Type	Production	Advantages	Limitations	Total Number of Vaccines	Leading Vaccines Name (Manufacturer)	Clinical Phase	Route of Immunization *	Efficacy
Live-attenuated	(I) serial passage of pathogenic virus in cell culture (II) growing the virus under unfavorable conditions (III) genetically alteration via key genes	Higher immunogenicity, strong and long-lasting immune responses	Risk of genetical instability and retrieving virulence, need for biosafety facilities	5	Meissa (Codagenix/Serum Institute of India)	Phase I/II	IN	-
Inactivated (killed)	Inactivation of the virus by heat, chemicals or radiation	Higher immunogenicity, no risk of infection	Reduced immune response, need for biosafety facilities, lower purity	21	Corona Vac (SinoVac)	Phase IV	IM	50.7%
					BBIBP-CorV (Sinopharm)	Phase III	IM	79.3%
					BBV152 (Bharat Biotech)	Phase III	IM	-
Vector vaccines	Non-pathogenic viral vectors delivering gene of viral antigens into the host cells	No risk of infection, no integration to host genome, strong in cellular and humoral Immune responses, fast to produce	Pre-immunity against the vector reducing vaccine efficacy, risk of adverse reactions	12 (Non-replicating) 6 (Replicating)	AZD1222 (AstraZeneca)	Phase IV	IM	70.4%
					JNJ78436735 (Johnson & Johnson)	Phase IV	IM	66%
					Ad5nCoV (CanSino Biologics)	Phase III	IM	65.3%
					Sputnik V (Gamaleya Research Institute)	Phase III	IM	91.6%
					flu-based-RBD (Jiangsu Provincial CDC)	Phase II	IN	-
					VSV-S (Israel Institute for Biological Research/ Weizmann Institute of Science)	Phase I/II	IM	-
Protein subunit	Recombinant synthesis of whole protein or its segment	No risk of infection, no risk of genome integration, targeted immune responses	Need for some booster doses and optimal adjuvant, reduced T-cell immunity	24	NVX-CoV2373 (Novavax)	Phase III	IM	89.7%
					ZF 2001 (Anhui Zhifei Longcom Biopharmaceutical)	Phase III	IM	-
					BP-COVID-19/KBP-201 (Kentucky Bioprocessing)	Phase III	IM	-
DNA	plasmid vector containing a gene of antigenic protein	Stimulation of humoral and cellular responses, no risk of infection, ease of production, stability at room temperature	Need for delivery vectors, electroporation and/or adjuvants to enhance their immunogenicity	11	INO-4800 (Inovio Pharmaceuticals)	Phase II/III	ID	-
					AG0301-COVID19 and AG0302-COVID19 (AnGes/Osaka University)	Phase II/III	IM	-
					ZyCoV-D (Cadila Healthcare Limited)	Phase III	ID	-
Virus-like particle (VLP)	Empty virus particles presenting several copies of the same antigen on their surface	No risk of infection, no viral genome	Challenging development and assembly process, reduced immunogenicity, Lower purity	2	Medicago Inc.	Phase III	IM	-
					SpyBiotech/Serum Institute of India	Phase II	IM	-
mRNA	mRNA of the antigenic protein encapsulated in lipid nanoparticles	Stimulation of humoral and cellular responses, No risk of infection, ease of production, no risk of genome integration	Need for delivery vectors, unstable, need for strict cold chain for distribution and storage	8	BNT16b2 (Pfizer/ BioNTech)	Phase IV	IM	95%
					mRNA-1273 (Moderna)	Phase IV	IM	94%
					CVnCOV (CureVac)	Phase III	IM	-

* IM: intramuscular; IN: intranasal.

**Table 2:** DMTs and COVID-19 infection: benefits or risks.

DMT Class	Mode of Action	Immuo-Suppressive?	Risk Category	Continue in Case of Infection?	Preventive Effects	Depletive Effects	Effect on Immune Responses	Time Window for Vaccination
IFN-β	Immunomodulatoy, pleitropic immune effects	No	Very low	Yes	Antiviral and anti-inflammatory by increasing levels of IL-10 and decreasing TNF-α, IFN-γ and IL-17	-	+IgG titers	Not neccessary
Glatiramer acetate	Immunomodulatoy, pleitropic immune effects	No	Very low	Yes	Anti-inflammatory, Prevents ARD via blocking TNF-α, IFN-γ and IL-12 and increasing IL-10 and IL-4	-	+IgG titers	Not neccessary
Teriflunomide	Dihydro-orotate dehydrogenase inhibitor, anti-proliferative	Possible (no well-defined	Very low	Yes	Antiviral	-	−/+ IgG titers	Not neccessary
Dimethyl fumarate	Pleotropic, NRF2 activation, downregulation of NFΚβ	Yes, continously	Low	Yes	Anti-oxidative, cytoprotective, antiviral and anti-inflammatory	Patients with a total lymphocyte count of <800/mm^3^ are at a higher risk of develping COVID-19 complications	+IgG titers	Maybe
Natalizumab	Anti-VLA4, selective adhesion molecule inhibitor	Yes, continoulys	Low	Yes or miss infusion depending on timing	-	May prolong viral shedding in mocus and gut	+IgG titers	NA
S1P modulators (Fingolimod, siponimod, ozanimod, ponesimod)	Selective S1P modulator, prevents egress of lymphocytes from lymph nodes	Yes, continously	Low	Yes or temporary suspension of dosing	Fingolimod under trial as anti-inflammmatory therapy for ARD	May prolong viral shedding	Fingolimod: −IgG titers	Not recommended
Anti-CD20 (Ocrelizumab, ofatumumab, Rituximab, ublituximab)	Anti-CD20 mAb: B-cell depleter	Yes, continously	Intermediate	Temporary suspension of dosing depending on timing	-	Particularly ocrelizumab may prolong viral shedding	Ocrelizumab: −IgG titers ofatumumab: +IgM and +IgG titers	12 weeks: ocrelizumab and rituximab 4 weeks: ofatumumab
Cladribine	Deoxyadenosine (purine) analogue, adenosine deaminase inhibitor, blocks T- and B-cell proliferation	Yes, intermittent	Intermediate	Temporary suspension of dosing depending on timing	-	Prolong viral shedding	−IgM and −IgG titers	4–6 weeks
Alemtuzumab	Anti-CD52 mAb: B- and T-cell depleter	Yes, intermittent	High *	Suspend dosing	-	Prolong viral shedding	NA	24 weeks
Mitoxantrone	Immune depleter, blocks IFN-γ, TNF-α and IL-2	Yes, intermittent	High *	Suspend dosing	-	Prolong viral shedding	NA	NA
Corticosteroids	Immune depleter	Yes, continously	High *	Suspend dosing	-	Prolong viral shedding	NA	4 weeks

* Risk of acquiring SARS-CoV-2 infection during the complete immunosuppression phase.

**Table 3:** Effect of DMTs on immune responses raised against different SARS-CoV-2 vaccine modules.

DMTs	Number of Cases	SARS-CoV-2 Vaccine	Sample	Immune Response	Detection Kit/Assay	Results	Reference
Fingolimod or ocrelizumab	32	BNT162b2 mRNA or mRNA-1273	Serum	Humoral	ELISA	Lower anti-Spike IgG (62.5%)	Guerrieri et al. [82]
Ocrelizumab or rituximab	20	SARS-CoV-2 mRNA vaccines	Plasma and PBMC	Humoral and cellular	ELISA, FACS	Lower anti-Spike IgG and anti-RBD IgG titers, robust antigen-specific CD4+ and CD8+ T-cell responses	Apostolidis et al. [83]
Rituximab or ocrelizumab	96	BNT162b2 mRNA or mRNA-1273	Serum, whole blood	Humoral and cellular	Anti S-protein IgG ELISA test from Euroimmun (Lübeck, Germany)	Lower anti-Spike IgG (49%), IFN-γ raised only in 20% patients	Moor et al. [84]
Ocrelizumab, rituximab, or fingolimod	473	BNT162b2 mRNA, Johnson and Johnson, or ChAdOx1 nCoV-19	Dried blood spot	Humoral	COVID-SeroKlir two-step ELISA (Kantaro Biosciences, USA) for detection of Anti-RBD IgG	Lower anti-RBD IgG	Tallantyre et al. [85]
Ocrelizumab	4	BNT162b2 mRNA	Serum	Humoral	LIAISON^®^ SARS-CoV-2 TrimericS IgG assay (DiaSorin S.p.A.,Saluggia, Italy), and CLIA) technology for the detection of IgG antibodies to trimeric spike protein (anti-TSPIgG), including neutralizing antibodies	Lower anti-Spike IgG (62.5%)	Gallo et al. [86]
Cladribine or ocrelizumab	2	BNT162b2 mRNA or AstraZeneca	Serum	Humoral	NA	Protective anti-spike IgG	Buttari et al. [87]
Rituximab	1	Gam-COVID-Vac	Serum	Humoral	ELISA	Lower anti-Spike IgG	Etemadifar et al. [88]
Cladribine, ocrelizumab, or fingolimod	125	BNT162b2 mRNA	Serum	Humoral	EUROIMMUN anti-SARS-CoV-2 IgG quantitative ELISA kit (EI, Lubeck, Germany) for detection of S1 subunit	Lower anti-spike IgG (22.7%) in Ocrelizumab group, no response in fingolimod group	Achiron et al. [89]
Natalizumab	26	BNT162b2 mRNA	Serum	Humoral	LIAISON^®^ SARS-CoV-2 TrimericSIgG assay (DiaSorin-S.p.A.)	Efficient short-term humoral response	Capuano et al. [90]
Cladribine, teriflunomide, ocrelizumab, rituximab, ofatumumab, fingolimod, ozanimod, cladribine, teriflunomide	120	BNT162b2 mRNA or mRNA-1273	Serum	Humoral	Chemiluminescence microparticle immunoassay (Abbott; quantification limits) IgG assay for detection of Anti- RBD Abs	Lower IgG levels in anti-CD20 mAbs and S1P modulators groups	Disanto et al. [73]
Anti-CD20 mAbs, S1P modulators, IFNβ-1a, teriflunomide, dimethyl fumarate or natalizumab	28	BNT162b2 mRNA or mRNA-1273	Serum	Humoral	Abbott or Roche SARS-CoV- 2 IgG assay for detection of Anti- spike protein Abs	Lower IgG levels in anti-CD20 mAbs and S1P modulators groups	Bigaut et al. [91]
Ocrelizumab or natalizumab	48	BNT162b2 mRNA or mRNA-1273	Serum, Whole blood	Humoral and cellular	Roche Elecsys Anti-SARS-CoV-2 S immunoassay and Adaptive Biotechnologies T-Detect COVID Test	Natalizumab-treated group produced both humoral and cellular responses, ocrelizumab- treated group were Ab negative but T-cell response positive	Katz et al. [92]
Anti-CD20 mAbs, S1P modulators, IFNβ-1a, IFNβ-1b, cladribine, teriflunomide, diroximel fumarate, dimethyl fumarate natalizumab, alemtuzumab	67	BNT162b2 mRNA, mRNA-1273, ChAdOx1nCoV-19	Serum	Humoral	Labcorp anti-SARS-CoV-2 semi-quantitative IgG ECLIA assay against the spike protein RBD	Lower Ab levels in anti-CD20 mAbs and S1P modulators groups	Conte et al. [93]
Glatiramer acetate	1	Heterologous strategy:ChAdOx1 nCoV-19/ mRNA BNT162b2	Serum	Humoral	LIAISON^®^ SARS-CoV-2 Trimeric S IgG assay (DiaSorin, Saluggia, Italy) and the Architect^®^ anti-spike test (Abbott, Rungis, France) against S-protein, iFlash^®^-2019-nCoV NAb (Orgentec^®^, Trappes, France) assay to measure neutralization antibodies	Strong anti-S antibody response and good neutralizing antibody response	Michiels et al. [94]

## Data Availability

Not applicable.
